# Evaluation of Smell Function in Patients with Childhood Epilepsy with Centrotemporal Spikes at a Pediatric Neurology Clinic—A Case–Control Study

**DOI:** 10.3390/jcm13216474

**Published:** 2024-10-29

**Authors:** Orhan Coşkun, Burçin Nazlı Karacabey, Afra Ünal, Samet Paksoy, Hale Nur Durak

**Affiliations:** 1 Department of Pediatric Neurology, Gaziosmanpaşa Training and Research Hospital, 34255 Istanbul, Turkey; afraunal@hotmail.com (A.Ü.); sametpaksoy@gmail.com (S.P.); hndurak@gmail.com (H.N.D.); 2 Department of Pediatric Neurology, Istanbul Faculty of Medicine, Istanbul University, 34093 Istanbul, Turkey; bnazlikaracabey@hotmail.com

**Keywords:** smell function, children, epilepsy with centrotemporal spikes

## Abstract

**Objective:** Childhood epilepsy with centrotemporal spikes (CECTS) is associated with cognitive, behavioral, and language difficulties. These epileptic discharges predominantly occur in the temporal lobe, which is known to be involved in olfactory functions. This study aims to assess olfactory dysfunction in patients with CECTS. **Methods:** This study included patients diagnosed with CECTS who were attending follow-ups at the Department of Child Neurology between January 2022 and July 2023. Olfactory function was evaluated using the Sniffin’ Sticks (Burghart GmbH, Wedel, Germany) 12-point screening test, which was administered to 44 patients and 35 controls. The smell test and the final control EEGs were performed simultaneously. **Results:** A total of 44 patients and 35 control subjects were enrolled in this study. The smell scores were significantly lower in the patient group compared to the control group (*p* = 0.029). The patient group had a very high prevalence of anosmia compared to the control group. The normosmia rate in the control group was significantly higher. No significant difference was observed in the smell scores based on EEG findings or antiepileptic drug type. **Conclusions:** Olfactory dysfunction was identified in patients with CECTS compared to healthy controls, yielding results consistent with findings for other types of epilepsy. Olfactory dysfunction was detected in a greater frequency among the patients diagnosed with CECTS than among the healthy control group, and similar results were obtained with other types of epilepsy. It was deduced that these patients may experience problems with smell sensitivity throughout their lives. The most important result of this study is that this condition should be taken into account in regard to patients’ well-being and lives.

## 1. Introduction

Rolandic epilepsy, also known as benign childhood epilepsy with centrotemporal spikes (CECTS), is the most prevalent epileptic syndrome in the idiopathic epilepsy group among children [1,2]. Clinical manifestations of this electroclinical syndrome, with an incidence reported to be between 15% and 24%, include unilateral facial motor sensory symptoms, oro-pharyngo-laryngeal symptoms, dysarthria, and increased salivation. The typical onset age for BECTS ranges from 2 to 14 years, peaking between 5 and 8 years [3]. Electroencephalograms of CECTS patients commonly reveal discharges in the centrotemporal region.

Despite being a benign form of epilepsy, studies have indicated associations with cognitive, behavioral, and language difficulties [4,5,6,7,8,9,10], although some studies suggest there is no correlation [11]. Early detection and management of such conditions are crucial for enhancing patients’ quality of life.

**Figure 1:**
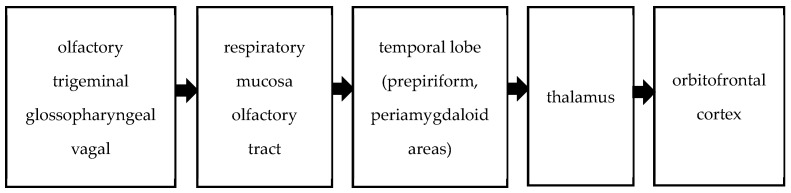
Olfactory pathway.

Olfactory stimuli are transmitted to the central nervous system via the olfactory, trigeminal, glossopharyngeal, and vagal nerves. The trigeminal, glossopharyngeal, and vagus nerves perceive odors through their free endings in the respiratory mucosa [12]. Central olfactory pathways involve the convergence of axons from bulb cells into the olfactory tract, extending to the primary olfactory cortex of the temporal lobe, comprising the prepiriform and periamygdaloid areas. These pathways project from the temporal lobe to the thalamus, ultimately reaching the orbitofrontal cortex. This network is summarized in Figure 1. Olfactory pathways form an intricate network within the central nervous system, influencing odor perception, behavior, memory, and eating choices [13,14]. Impairments in olfactory and identification performance have been observed in patients with temporal lobe epilepsy [15,16]. As mentioned above, the sense of smell involves multiple areas of the brain.

It is thought that the discharges observed in the centrotemporal regions in patients with CECTS could lead to olfactory dysfunction similar to that seen in temporal lobe epilepsies. Smell identification tests often utilize odors presented in glass bottles or scent-impregnated paper. Participants are tasked with recognizing various scents, commonly assessed using the “Sniffin’ Sticks” test [17].

As observed in the electroencephalograms of patients with CECTS, epileptic discharges are seen spreading to the central temporal and sometimes frontal regions of the brain. This pathway is among those responsible for olfaction, as described above. A pathology that develops in this region can affect many functions. Olfactory dysfunction has been demonstrated in epilepsies affecting the temporal region. The types of pathologies that this disease, for which no atrophy or structural anomalies can be seen in cranial imaging and whose genetic causes are unknown, may cause are still being investigated. Olfactory dysfunction in these patients has never been investigated before. This study aims to assess olfactory function in CECTS patients, recognizing that an accompanying olfactory dysfunction could impact behavior and quality of life.

## 2. Materials and Methods

### 2.1. Participants

Patients diagnosed with CECTS who were followed up at the department of child health and diseases of the department of child neurology between January 2022 and July 2023 constituted our study group. Children and adolescents aged 6–16 years with CECTS were included in this study if they exhibited typical seizure semiology, benign epileptic discharges as defined by the International League Against Epilepsy, unremarkable brain magnetic resonance imaging findings, and normal neurological examination results [1].

Patients with known comorbidities, chronic neuropsychiatric diseases, or allergies that could affect the result of the smell test; who used any medication other than antiepileptics; or who used multiple antiepileptic medications were excluded from this study. All patients and control groups underwent examination by an ear, nose, and throat specialist before being tested in the pediatric clinic. No adverse conditions were detected, and there were no complaints made. None of the participants had any allergies or other ailments that could cause hyposmia. There was no growth-and-development delay in any of the patients or control group members. The height and weight percentiles of all patients were found to be between the 25th and 75th percentiles. This rules out Kallmann syndrome associated with hypogonadism. Additionally, none of the participants had a history of COVID-19 in the past 1.5 years. There were no participants in our study who reported having any prior olfactory dysfunction. Furthermore, the brain MRIs taken during the patients’ follow-ups did not show any signs of septal deviation.

**Figure 2:**
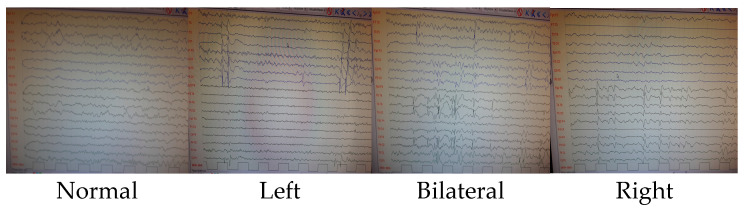
EEG findings.

Patients who showed improvement via an electroencephalogram at the last follow-up were also included in this study. The smell test and the final control EEGs were performed simultaneously. The duration between the patients’ first seizures and the time this study was conducted varies from 1 month to 4.2 years. Rather than focusing on worsening over time, the correlation with EEG findings was examined. It was classified according to the last recorded EEG findings based on whether prominent sharp slow-wave activity in the left hemisphere, prominent activity in the right hemisphere, or significant activity in both hemispheres was shown. Patients whose findings normalized in their last EEGs were classified as normal. See Figure 2 for further details.

**Figure 3:**
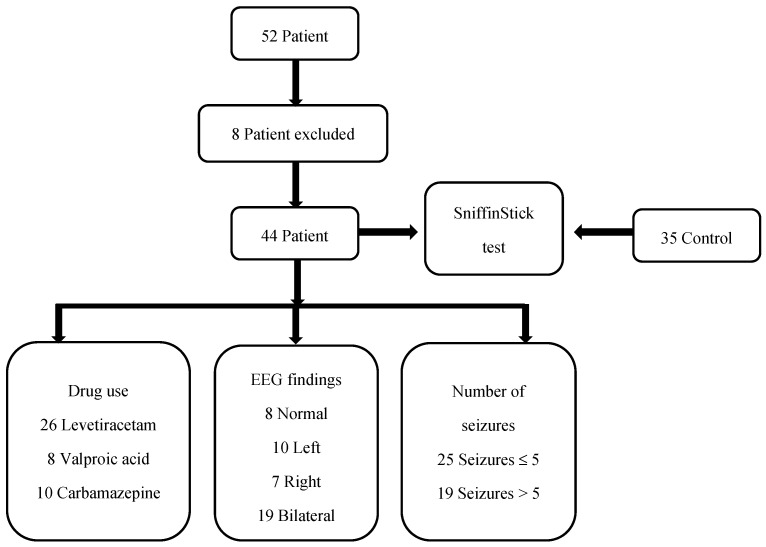
The stages of this study.

At the regular follow-ups, there were 52 patients diagnosed with CECTS. Three patients could not be reached for pre-test EEG recording. Two patients had a low school-grade average. Three patients were using a combination of two antiepileptic medications. In total, 44 patients were included in this study. The stages of this study are presented in Figure 3.

The study protocol was approved by the ethics committee of the research hospital. All participants and/or their legal representatives provided signed written consent forms.

### 2.2. Assessment of Olfactory Function

**Figure 4:**
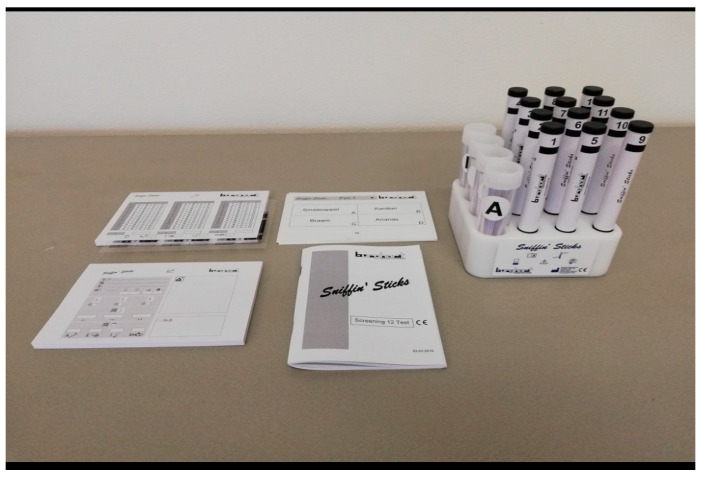
Sniffin’ Sticks test.

To assess olfactory function, we utilized the 12-item Sniffin’ Sticks test scent sticks (Burghart GmbH, Wedel, Germany). Each stick was presented to the participant for 3–4 s at equal distances from both nostrils and at a distance of 2 cm. Patients were then asked to identify the correct scent from among the four options provided. There was a 30 s break between presenting each scent. Participants were instructed not to consume any food or drink, except water, and refrain from smoking for 15 min prior to the test. The examiner did not wear gloves during the administration of the test. Following the completion of all 12 questions, a score ranging from 0 to 12 was obtained, and participants were categorized based on percentiles (normosmia, hyposmia, anosmia) [17]. A photograph of the equipment used in the test is presented in Figure 4.

The control group was matched for age, sex, and socioeconomic status and comprised healthy children without any known acute or chronic illnesses. Individuals with similar academic achievements to those of the patient group were selected for the control group.

Participants with low academic performance were excluded from both the patient and control groups. The overall grade point average of all participants was deemed satisfactory compared to national standards. While the test utilized in our study was standardized, variations may occur depending on economic and sociocultural factors. Therefore, a control group was included to account for potential confounders. Children’s unfamiliarity with the tropical fruits included in the test could have biased the results. Consequently, the control group was selected from individuals with similar sociocultural backgrounds in the region. Thus, relying solely on test standardization for decision-making may be misleading.

## 3. Statistical Analysis

During the evaluation of the findings obtained in this study, IBM Statistical Package for Social Sciences (SPSS) software package (Version 23, Chicago, IL, USA, 2015) was utilized for statistical analysis. In addition to descriptive statistical methods (mean, median, standard deviation, and frequency), an independent samples *t*-test was used for comparing parameters with a normal distribution, while the Kruskal–Wallis test was employed for parameters with a non-normal distribution. Pearson’s chi-squared test was employed to compare categorical variables. Significance was set to *p* < 0.05.

## 4. Results

The demographic data is provided in Table 1. Upon comparing the smell scores between the patient and control groups, it was observed that the smell scores in the control group were significantly higher than those in the patient group (*p* = 0.029) (Table 2). Each subscript letter denotes a subset of patient and control group categories whose column proportions do not significantly differ from each other at a significance level of *p* < 0.05. In each row, each possible pair of percentages was compared using the z-test. Similar subscripts indicate no difference. Statistically significant differences were observed in smell classes when comparing the patient and control groups (χ2(2) = 22.116, *p* < 0.01). The anosmia smell class included significantly more children in the patient group as opposed to the control group, while the normosmia smell class included significantly more children in the control group as opposed to the patient group (Table 3).

Upon comparing the smell scores among the children in the patient group, which was divided into three groups (valproic acid, levetiracetam, carbamazepine) based on the drugs they used, no statistically significant difference was observed among the three groups (*p* > 0.05) (Table 4).

No statistically significant difference was observed when comparing smell scores among the children in the patient group, which was divided into four groups (normal, left, right, and bilateral) based on the EEG findings (*p* > 0.05) (Table 5). When comparing smell scores among the children in the patient group, which was divided into two groups (≤5 and >5) based on the total number of clinical seizures, no statistically significant difference was observed (*p* > 0.05) (see Table 6).

## 5. Discussion

The study aimed to investigate whether the olfactory acuity of patients with CECTS differs from that of the normal population. In the electroencephalograms of the patients with CECTS, epileptic discharges spreading to the central temporal and sometimes frontal regions of the brain can be seen. This pathway is one of the ones responsible for olfaction. Therefore, it has been considered that olfactory dysfunction may accompany this condition.

Attention deficit hyperactivity disorder (20%), mood disorders (23.6%), learning disabilities (14.5%), and behavioral disorders (7.3%) were identified among patients with CECTS. Furthermore, it has been noted that patients experience various difficulties in verbal perception and promoter performance [5]. Despite being a benign form of epilepsy, numerous dysfunctions have been identified in various studies [6,7,8,9,10]. Studies on cognitive disorders are commonly reported in the literature.

Olfactory pathways constitute a widespread and intricate network in the central nervous system, influencing a multitude of choices such as odor perception, behavior, memory, and eating [13,14]. Hence, we aimed to investigate olfactory dysfunction in CECTS patients.

During olfactory processing, secondary processing occurs in the orbitofrontal, mesial temporal, thalamic, and hypothalamic regions, and dysfunction has also been identified following temporal lobe resection [18,19]. The relationship between left cerebral structures, active in emotional processes, and olfactory function has been demonstrated using imaging methods [20]. Studies have demonstrated impaired olfactory and identification performance among patients with temporal lobe epilepsy. These studies have reported lower performance in right-temporal-lobe epilepsy or dysfunction regardless of the lobe [15,16]. Additionally, a study examining olfactory auras in temporal lobe epilepsies reported a frequency of 5.5%, suggesting a potential association with mesial temporal structures [21].

In our study, it was found that the smell scores were significantly lower in the epilepsy group according to the results of the Sniffin’ Sticks (Burghart GmbH in Wedel, Germany) 12-point screening test (*p* < 0.05). These scores are classified as anosmia, hyposmia, and normosmia based on the test scale, with values varying according to age and sex, and standardized. However, tropical fruits are included in the scents presented during this test, which may prevent children unfamiliar with these fruits from correctly performing the test. The control group was selected from among children studying in the same region due to their access to similar foods. These children, belonging to the same economic income group, are familiar with similar smells.

Upon examining the results, it was found that the number of children in the anosmia smell class was significantly higher in the patient group than in the control group. In regard to the normosmia smell class, the number of children in the control group was significantly higher than that in the patient group. These findings indicate olfactory dysfunction in children with CECTS. A result parallel to the results obtained in other temporal lobe epilepsy studies was obtained, and this is the first study on this subject. The reason we questioned whether this situation was related to perception was that the school performances of the two groups were similar. However, previous studies indicating cognitive dysfunction in this patient group may have influenced this finding.

The effect of disorders in the temporal region on olfactory function is known. It is believed that epileptic activity occurring in this region has negative effects on olfactory function. As we assessed patients using different types of medications, when we compared them, no difference was detected in smell acuity depending on the medications used (*p* > 0.05). Various side effects of these drugs are known. However, the effect of the drugs used in our study on the results of the patient group could not be tested. In addition, no significant difference was detected in the EEG findings when the smell test was performed. There was no difference in the smell test results between patients whose EEG results were normal at the time of the test and those with distorted EEG results. The fact that no difference was detected in the olfactory functions of patients with improved electroencephalogram results made us think that this mechanism is more complex. The reason EEGs were used in the follow-ups was to observe this situation. The smell scores of patients with more and fewer than five clinical seizures were compared to the risk of differences in the number of seizures. Additionally, it was observed that the number of clinical seizures had no correlation with olfactory dysfunction. For the correct functioning of the olfactory function, the temporal lobe, amygdala, orbitofrontal cortex, and many other regions must be involved. It is surprising to find these results in the context of CECTS, which is generally considered a benign type of epilepsy.

In this group, wherein behavioral problems and cognitive disorders are generally described, olfactory dysfunction was also observed, perhaps due to the same mechanism. Olfactory dysfunction can affect the quality of life of these children and may lead to nutritional disorders [22]. However, in a previous study we conducted on this topic, no differences in eating behaviors were observed. There were also no height or weight differences between the patient and control groups [11].

Nevertheless, olfactory dysfunction may have negative effects on the well-being of patients. Problems in odor discrimination can lead to many issues in humans regarding danger avoidance, mate selection, and memory recall. For this reason, olfactory function is critically important for humans. This condition could also contribute to the psychiatric problems observed in these patients. Additionally, this condition may also create difficulties in terms of the patients’ social relationships. In our study, there were no patients with cognitive impairment. None of our patients were being followed by the psychiatry department for behavioral disorders. As the olfactory pathways are connected with memory, nutrition, and behavioral regions, they may play a role in the mechanisms of the disorders observed in patients with CECTS. The lifelong continuity of this condition is unknown. Studies related to the adult lives of patients whose seizures have improved are limited. Therefore, more such studies need to be conducted.

Our exclusion of any patients with cognitive impairment indicates that we were unable to assess olfactory function in this group. The aim here was to prevent perceptual disturbances from affecting the study’s results. However, the limitation in this regard is that we were unable to evaluate olfactory function in these patients. Olfactory dysfunction may be more pronounced in individuals with cognitive impairment. Since our study was conducted on a specific patient group, the conditions in other childhood epilepsies are unknown. This situation may be critically important with respect to the mechanisms of similar behavioral disorders in these patients. Research can be conducted on larger patient groups and the effects of childhood epilepsies in adulthood. Different studies are needed to determine whether this situation is temporary or permanent in later years of life.

## 6. Conclusions

Although CECTS is a benign form of epilepsy, it can induce olfactory dysfunction, as with other forms of epilepsy, alongside behavioral issues. The smell scores in the control group were significantly higher than those in the patient group. Statistically significant differences were observed in terms of smell classes. The anosmia smell class was significantly more prominent in the patient group. Olfactory dysfunction was observed within the entire patient cohort irrespective of EEG findings, clinical seizure count, and medication regimens. The olfactory mechanism is intricate, and assessing distinct epilepsy types separately may yield more precise outcomes.

The most important finding of this study is that olfactory dysfunction may have negative effects on the corresponding patients’ well-being. This condition can impact their lives and may be one of the underlying factors of various behavioral disorders demonstrated in previous studies. While I do not believe it is necessary to routinely conduct odor tests on all patients with CECTS, it is important to be aware that such comorbid conditions may coexist.

## Figures and Tables

**Table 1:** Demographic findings—smell percentiles.

**Total number (*n*, %)**	**79 (%100)**
Patient	44 (%55.7)
Control	35 (%44.3)
**Age (year) (mean ± SD)**	**10.61 ± 3.04**
Patient	9.82 ± 2.62
Control	11.60 ± 3.27
**Gender for patient group (*n*, %)**	**44 (%100)**
Female	23 (%52.3)
Male	21 (%47.7)
**Gender for control group (*n*, %)**	**35 (%100)**
Female	14 (%40)
Male	21 (%60)
**Smell score (mean ± SD)**	**7.80 ± 2.36**
Patient	6.80 ± 2.38
Control	9.06 ± 1.64
**Smell classes for patient group (*n*, %)**	**44 (%100)**
Anosmia	20 (%45.5)
Hyposmia	17 (%38.6)
Normosmia	7 (%15.9)
**Smell classes for control group (*n*, %)**	**35 (%100)**
Anosmia	1 (%45.5)
Hyposmia	15 (%38.6)
Normosmia	19 (%15.9)
**Smell percentiles for patient group (*n*, %)**	**44 (%100)**
Under 10	24 (%54.5)
10	9 (%20.5)
Between 10 and 50	6 (%13.6)
50	3 (%6.8)
Between 50 and 90	2 (%4.5)
90	0 (%0)
Above 90	0 (%0)
**Smell percentiles for control group (*n*, %)**	**35 (%100)**
Under 10	4 (%11.4)
10	9 (%25.7)
Between 10 and 50	11 (%31.4)
50	4 (%11.4)
Between 50 and 90	2 (%5.7)
90	4(%11.4)
Above 90	1 (%2.9)
**Drug use for the patient group (*n*, %)**	**44 (%100)**
Levetiracetam	26 (%32.9)
Valproic acid	8 (%10.1)
Carbamazepine	10 (%12.7)
**Latest EEG findings for the patient group (*n*, %)**	**44 (%100)**
Normal	8 (%10.1)
Left	10 (%12.7)
Right	7 (%8.9)
Bilateral	19 (%24.1)
**Number of seizures for patient group (*n*, %)**	**44 (%100)**
5 and under	25 (%31.6)
Above 5	19 (%24.1)
**Disease duration for patient group** **(month) (mean ± SD)**	**38.5 ± 37.28**

*n*: Number of patients; mean ± SD: mean ± standard deviation.

**Table 2:** Comparison of the patient group and the control group in terms of smell scores.

	Patient	Control	*p* ^a^
	Group	Group	
Smell score (mean ± SD)	6.80 ± 2.38	9.06 ± 1.64	0.029

^a^ independent samples *t*-test; mean ± SD: mean ± standard deviation.

**Table 3:** Comparison of patient group and control group in terms of smell classes.

	Patient	Control	Total
Anosmia (*n*, %)	20 _a_	1 _b_	21
	%95.2	%4.8	%100
Hyposmia (*n*, %)	17 _a_	15 _a_	32
	%53.1	%46.9	%100
Normosmia (*n*,%)	7 _a_	19 _b_	26
	%26.9	%73.1	%100
Total (*n*, %)	44	35	79
	%55.7	%44.3	%100

Pearson’s chi-squared test and z-test (adjusted *p*-values Bonferroni method) were used. Each subscript letter indicates a subset of patient and control group categories that are not significantly different from each other at the column ratios, *p* < 0.05 significance level. Each row compares every possible percentage pair using a z-test. If they are not different, they receive a similar subscript.

**Table 4:** Smell scores according to the drugs used in the patient group.

	Valproic Acid	Levetiracetam	Carbamazepine	*p*
Smell score (mean ± SD); (median)	6.25 ± 3.24; (6)	7.23 ± 2.39; (7.5)	6.10 ± 1.29; (5.5)	^a^ *p* = 0.240

^a^ Kruskal–Wallis test; mean ± SD: mean ± standard deviation.

**Table 5:** Smell scores of children in the patient group according to their latest EEG findings.

	Normal	Left	Right	Bilateral	*p*
Smell score (mean ± SD); (median)	6.25 ± 3.24; (6)	7.23 ± 2.39; (7.5)	6.10 ± 1.29; (5.5)	6.10 ± 1.29; (5.5)	^a^ *p* = 0.629

^a^ Kruskal–Wallis test; mean ± SD: mean ± standard deviation.

**Table 6:** Smell scores according to the number of seizures of children in the patient group.

	5 and Under	Above 5	*p* ^a^
Smell score (mean ± SD); (median)	7.32 ± 2.39	6.11 ± 2.23	0.708

^a^ Independent samples *t*-test; mean ± SD: mean ± standard deviation.

## Data Availability

The data associated with the paper are not publicly available but are available from the corresponding author on reasonable request.
